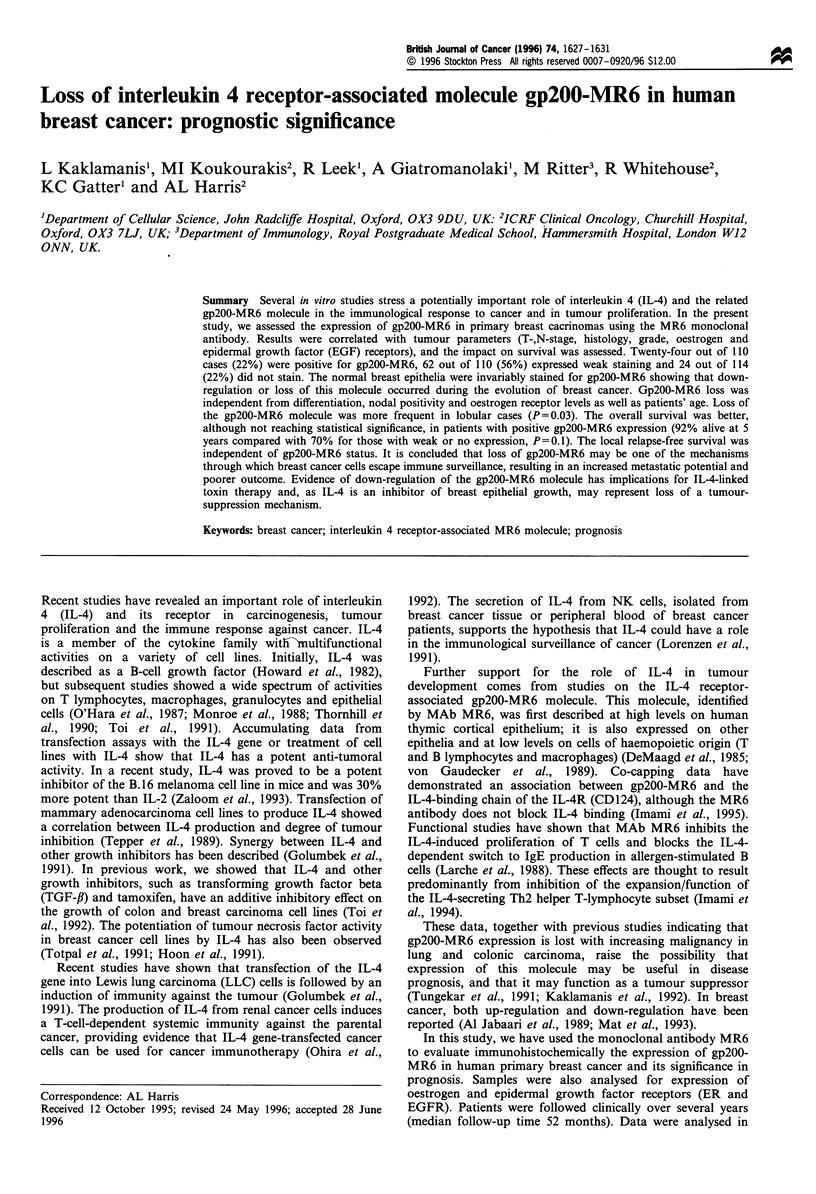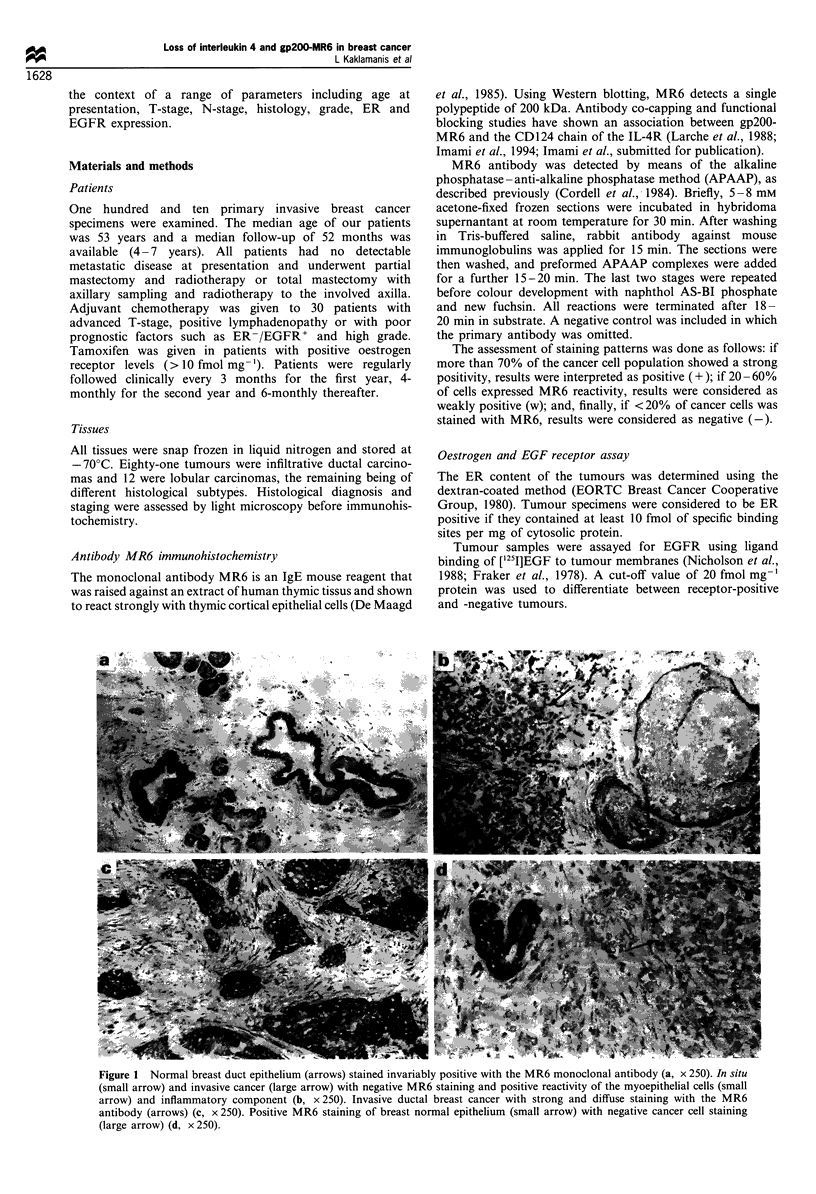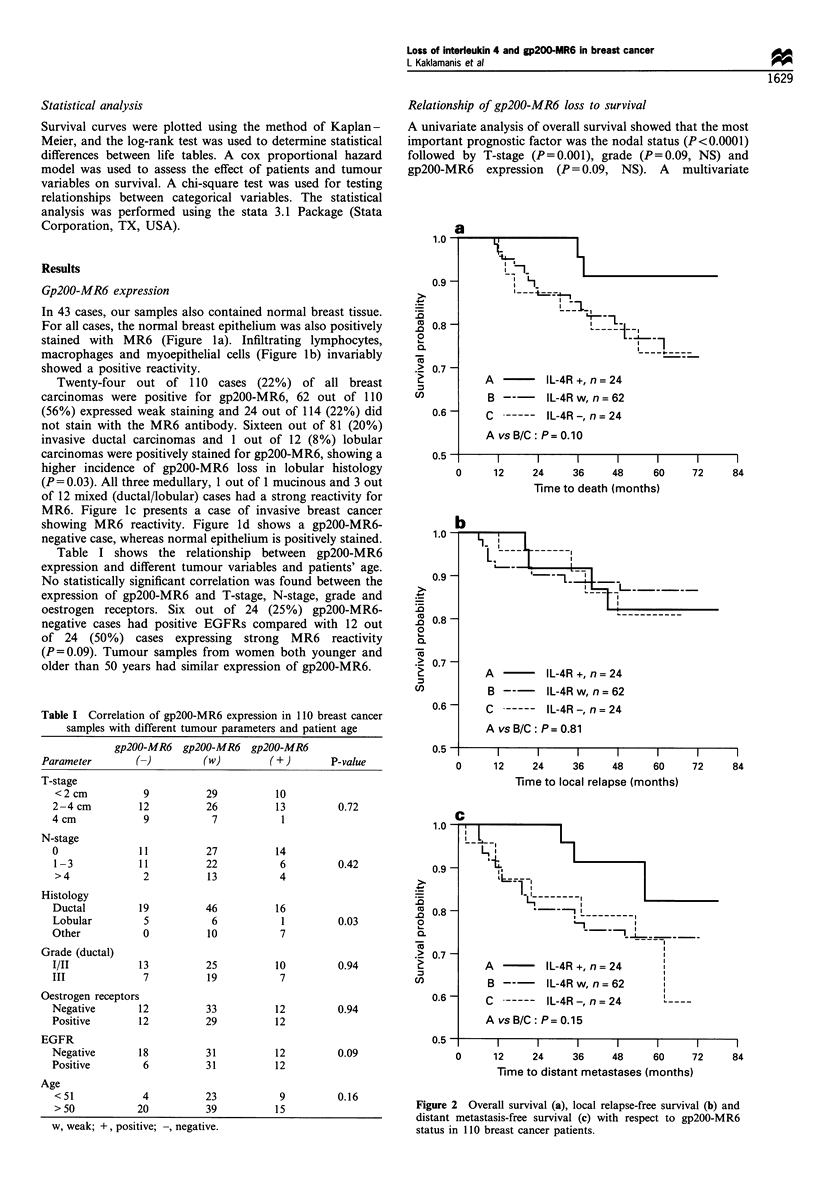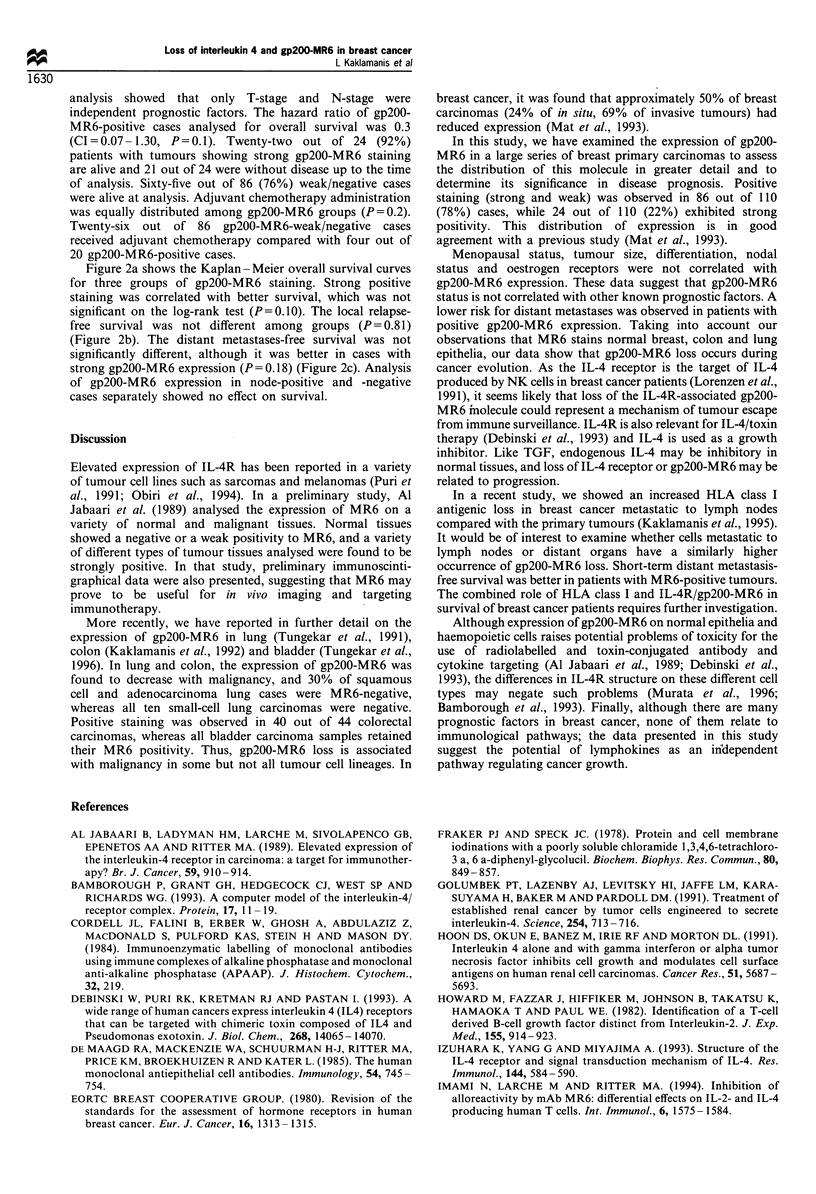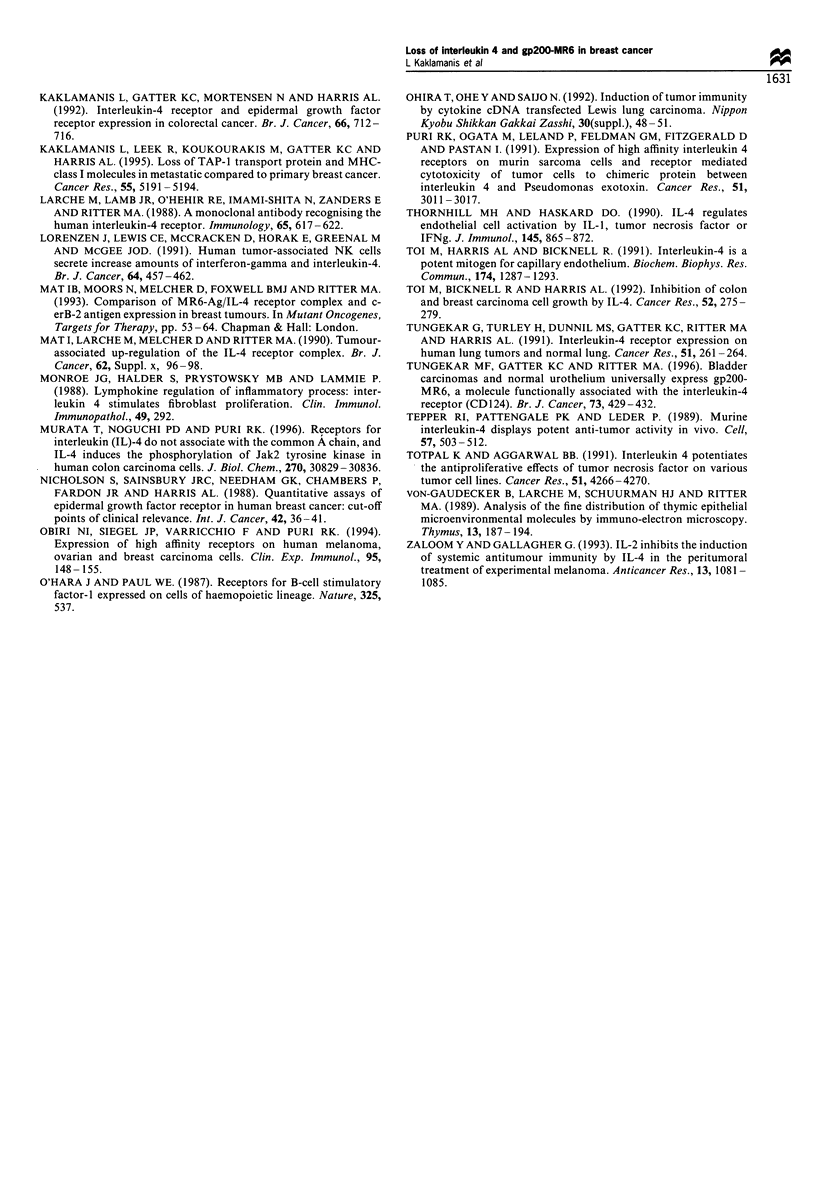# Loss of interleukin 4 receptor-associated molecule gp200-MR6 in human breast cancer: prognostic significance.

**DOI:** 10.1038/bjc.1996.599

**Published:** 1996-11

**Authors:** L. Kaklamanis, M. I. Koukourakis, R. Leek, A. Giatromanolaki, M. Ritter, R. Whitehouse, K. C. Gatter, A. L. Harris

**Affiliations:** Department of Cellular Science, John Radcliffe Hospital, Oxford, UK.

## Abstract

**Images:**


British Journal of Cancer (1996) 74, 1627-1631
© 1996 Stockton Press All rights reserved 0007-0920/96 $12.00

### Loss of interleukin 4 receptor-associated molecule gp200-MR6 in human breast cancer: prognostic significance

L Kaklamanis[1], MI Koukourakis[2], R Leek[1], A Giatromanolaki[1], M Ritter[3], R Whitehouse[2], KC Gatter[1] and AL Harris[2]

[1]*Department of Cellular Science, John Radcliffe Hospital, Oxford, OX3 9DU, UK:* [2]*ICRF Clinical Oncology, Churchill Hospital, Oxford, OX3 7LJ, UK;* [3]*Department of Immunology, Royal Postgraduate Medical School, Hammersmith Hospital, London W12 ONN, UK.*

**Summary** Several *in vitro* studies stress a potentially important role of interleukin 4 (IL-4) and the related gp200-MR6 molecule in the immunological response to cancer and in tumour proliferation. In the present study, we assessed the expression of gp200-MR6 in primary breast cacrinomas using the MR6 monoclonal antibody. Results were correlated with tumour parameters (T-,N-stage, histology, grade, oestrogen and epidermal growth factor (EGF) receptors), and the impact on survival was assessed. Twenty-four out of 110 cases (22%) were positive for gp200-MR6, 62 out of 110 (56%) expressed weak staining and 24 out of 114 (22%) did not stain. The normal breast epithelia were invariably stained for gp200-MR6 showing that down-regulation or loss of this molecule occurred during the evolution of breast cancer. Gp200-MR6 loss was independent from differentiation, nodal positivity and oestrogen receptor levels as well as patients' age. Loss of the gp200-MR6 molecule was more frequent in lobular cases ($P=0.03$). The overall survival was better, although not reaching statistical significance, in patients with positive gp200-MR6 expression (92% alive at 5 years compared with 70% for those with weak or no expression, $P=0.1$). The local relapse-free survival was independent of gp200-MR6 status. It is concluded that loss of gp200-MR6 may be one of the mechanisms through which breast cancer cells escape immune surveillance, resulting in an increased metastatic potential and poorer outcome. Evidence of down-regulation of the gp200-MR6 molecule has implications for IL-4-linked toxin therapy and, as IL-4 is an inhibitor of breast epithelial growth, may represent loss of a tumour-suppression mechanism.

**Keywords:** breast cancer; interleukin 4 receptor-associated MR6 molecule; prognosis

Recent studies have revealed an important role of interleukin 4 (IL-4) and its receptor in carcinogenesis, tumour proliferation and the immune response against cancer. IL-4 is a member of the cytokine family with multifunctional activities on a variety of cell lines. Initially, IL-4 was described as a B-cell growth factor (Howard *et al.*, 1982), but subsequent studies showed a wide spectrum of activities on T lymphocytes, macrophages, granulocytes and epithelial cells (O'Hara *et al.*, 1987; Monroe *et al.*, 1988; Thornhill *et al.*, 1990; Toi *et al.*, 1991). Accumulating data from transfection assays with the IL-4 gene or treatment of cell lines with IL-4 show that IL-4 has a potent anti-tumoral activity. In a recent study, IL-4 was proved to be a potent inhibitor of the B.16 melanoma cell line in mice and was 30% more potent than IL-2 (Zaloom *et al.*, 1993). Transfection of mammary adenocarcinoma cell lines to produce IL-4 showed a correlation between IL-4 production and degree of tumour inhibition (Tepper *et al.*, 1989). Synergy between IL-4 and other growth inhibitors has been described (Golumbek *et al.*, 1991). In previous work, we showed that IL-4 and other growth inhibitors, such as transforming growth factor beta (TGF-$\beta$) and tamoxifen, have an additive inhibitory effect on the growth of colon and breast carcinoma cell lines (Toi *et al.*, 1992). The potentiation of tumour necrosis factor activity in breast cancer cell lines by IL-4 has also been observed (Totpal *et al.*, 1991; Hoon *et al.*, 1991).

Recent studies have shown that transfection of the IL-4 gene into Lewis lung carcinoma (LLC) cells is followed by an induction of immunity against the tumour (Golumbek *et al.*, 1991). The production of IL-4 from renal cancer cells induces a T-cell-dependent systemic immunity against the parental cancer, providing evidence that IL-4 gene-transfected cancer cells can be used for cancer immunotherapy (Ohira *et al.*, 1992). The secretion of IL-4 from NK cells, isolated from breast cancer tissue or peripheral blood of breast cancer patients, supports the hypothesis that IL-4 could have a role in the immunological surveillance of cancer (Lorenzen *et al.*, 1991).

Further support for the role of IL-4 in tumour development comes from studies on the IL-4 receptor-associated gp200-MR6 molecule. This molecule, identified by MAb MR6, was first described at high levels on human thymic cortical epithelium; it is also expressed on other epithelia and at low levels on cells of haemopoietic origin (T and B lymphocytes and macrophages) (DeMaagd *et al.*, 1985; von Gaudecker *et al.*, 1989). Co-capping data have demonstrated an association between gp200-MR6 and the IL-4-binding chain of the IL-4R (CD124), although the MR6 antibody does not block IL-4 binding (Imami *et al.*, 1995). Functional studies have shown that MAb MR6 inhibits the IL-4-induced proliferation of T cells and blocks the IL-4-dependent switch to IgE production in allergen-stimulated B cells (Larche *et al.*, 1988). These effects are thought to result predominantly from inhibition of the expansion/function of the IL-4-secreting Th2 helper T-lymphocyte subset (Imami *et al.*, 1994).

These data, together with previous studies indicating that gp200-MR6 expression is lost with increasing malignancy in lung and colonic carcinoma, raise the possibility that expression of this molecule may be useful in disease prognosis, and that it may function as a tumour suppressor (Tungekar *et al.*, 1991; Kaklamanis *et al.*, 1992). In breast cancer, both up-regulation and down-regulation have been reported (Al Jabaari *et al.*, 1989; Mat *et al.*, 1993).

In this study, we have used the monoclonal antibody MR6 to evaluate immunohistochemically the expression of gp200-MR6 in human primary breast cancer and its significance in prognosis. Samples were also analysed for expression of oestrogen and epidermal growth factor receptors (ER and EGFR). Patients were followed clinically over several years (median follow-up time 52 months). Data were analysed in

Correspondence: AL Harris
Received 12 October 1995; revised 24 May 1996; accepted 28 June 1996

the context of a range of parameters including age at presentation, T-stage, N-stage, histology, grade, ER and EGFR expression.

### Materials and methods

### *Patients*

One hundred and ten primary invasive breast cancer specimens were examined. The median age of our patients was 53 years and a median follow-up of 52 months was available (4–7 years). All patients had no detectable metastatic disease at presentation and underwent partial mastectomy and radiotherapy or total mastectomy with axillary sampling and radiotherapy to the involved axilla. Adjuvant chemotherapy was given to 30 patients with advanced T-stage, positive lymphadenopathy or with poor prognostic factors such as $ER^-/EGFR^+$ and high grade. Tamoxifen was given in patients with positive oestrogen receptor levels ($>10$ fmol $mg^{-1}$). Patients were regularly followed clinically every 3 months for the first year, 4-monthly for the second year and 6-monthly thereafter.

### *Tissues*

All tissues were snap frozen in liquid nitrogen and stored at −70°C. Eighty-one tumours were infiltrative ductal carcinomas and 12 were lobular carcinomas, the remaining being of different histological subtypes. Histological diagnosis and staging were assessed by light microscopy before immunohistochemistry.

### *Antibody MR6 immunohistochemistry*

The monoclonal antibody MR6 is an IgE mouse reagent that was raised against an extract of human thymic tissus and shown to react strongly with thymic cortical epithelial cells (De Maagd *et al.*, 1985). Using Western blotting, MR6 detects a single polypeptide of 200 kDa. Antibody co-capping and functional blocking studies have shown an association between gp200-MR6 and the CD124 chain of the IL-4R (Larche *et al.*, 1988; Imami *et al.*, 1994; Imami *et al.*, submitted for publication).

MR6 antibody was detected by means of the alkaline phosphatase–anti-alkaline phosphatase method (APAAP), as described previously (Cordell *et al.*, 1984). Briefly, 5–8 mM acetone-fixed frozen sections were incubated in hybridoma supernantant at room temperature for 30 min. After washing in Tris-buffered saline, rabbit antibody against mouse immunoglobulins was applied for 15 min. The sections were then washed, and preformed APAAP complexes were added for a further 15–20 min. The last two stages were repeated before colour development with naphthol AS-BI phosphate and new fuchsin. All reactions were terminated after 18–20 min in substrate. A negative control was included in which the primary antibody was omitted.

The assessment of staining patterns was done as follows: if more than 70% of the cancer cell population showed a strong positivity, results were interpreted as positive (+); if 20–60% of cells expressed MR6 reactivity, results were considered as weakly positive (w); and, finally, if $<20\%$ of cancer cells was stained with MR6, results were considered as negative (−).

### *Oestrogen and EGF receptor assay*

The ER content of the tumours was determined using the dextran-coated method (EORTC Breast Cancer Cooperative Group, 1980). Tumour specimens were considered to be ER positive if they contained at least 10 fmol of specific binding sites per mg of cytosolic protein.

Tumour samples were assayed for EGFR using ligand binding of $[^{125}I]$EGF to tumour membranes (Nicholson *et al.*, 1988; Fraker *et al.*, 1978). A cut-off value of 20 fmol $mg^{-1}$ protein was used to differentiate between receptor-positive and -negative tumours.

**Figure 1** Normal breast duct epithelium (arrows) stained invariably positive with the MR6 monoclonal antibody (**a**, ×250). *In situ* (small arrow) and invasive cancer (large arrow) with negative MR6 staining and positive reactivity of the myoepithelial cells (small arrow) and inflammatory component (**b**, ×250). Invasive ductal breast cancer with strong and diffuse staining with the MR6 antibody (arrows) (**c**, ×250). Positive MR6 staining of breast normal epithelium (small arrow) with negative cancer cell staining (large arrow) (**d**, ×250).

*Statistical analysis*

Survival curves were plotted using the method of Kaplan–Meier, and the log-rank test was used to determine statistical differences between life tables. A cox proportional hazard model was used to assess the effect of patients and tumour variables on survival. A chi-square test was used for testing relationships between categorical variables. The statistical analysis was performed using the stata 3.1 Package (Stata Corporation, TX, USA).

### Results

*Gp200-MR6 expression*

In 43 cases, our samples also contained normal breast tissue. For all cases, the normal breast epithelium was also positively stained with MR6 (Figure 1a). Infiltrating lymphocytes, macrophages and myoepithelial cells (Figure 1b) invariably showed a positive reactivity.

Twenty-four out of 110 cases (22%) of all breast carcinomas were positive for gp200-MR6, 62 out of 110 (56%) expressed weak staining and 24 out of 114 (22%) did not stain with the MR6 antibody. Sixteen out of 81 (20%) invasive ductal carcinomas and 1 out of 12 (8%) lobular carcinomas were positively stained for gp200-MR6, showing a higher incidence of gp200-MR6 loss in lobular histology ($P=0.03$). All three medullary, 1 out of 1 mucinous and 3 out of 12 mixed (ductal/lobular) cases had a strong reactivity for MR6. Figure 1c presents a case of invasive breast cancer showing MR6 reactivity. Figure 1d shows a gp200-MR6-negative case, whereas normal epithelium is positively stained.

Table I shows the relationship between gp200-MR6 expression and different tumour variables and patients' age. No statistically significant correlation was found between the expression of gp200-MR6 and T-stage, N-stage, grade and oestrogen receptors. Six out of 24 (25%) gp200-MR6-negative cases had positive EGFRs compared with 12 out of 24 (50%) cases expressing strong MR6 reactivity ($P=0.09$). Tumour samples from women both younger and older than 50 years had similar expression of gp200-MR6.

**Table I** Correlation of gp200-MR6 expression in 110 breast cancer samples with different tumour parameters and patient age

| *Parameter* | *gp200-MR6 (−)* | *gp200-MR6 (w)* | *gp200-MR6 (+)* | P-*value* |
|---|---|---|---|---|
| T-stage | | | | |
| < 2 cm | 9 | 29 | 10 | |
| 2–4 cm | 12 | 26 | 13 | 0.72 |
| 4 cm | 9 | 7 | 1 | |
| N-stage | | | | |
| 0 | 11 | 27 | 14 | |
| 1–3 | 11 | 22 | 6 | 0.42 |
| > 4 | 2 | 13 | 4 | |
| Histology | | | | |
| Ductal | 19 | 46 | 16 | |
| Lobular | 5 | 6 | 1 | 0.03 |
| Other | 0 | 10 | 7 | |
| Grade (ductal) | | | | |
| I/II | 13 | 25 | 10 | 0.94 |
| III | 7 | 19 | 7 | |
| Oestrogen receptors | | | | |
| Negative | 12 | 33 | 12 | 0.94 |
| Positive | 12 | 29 | 12 | |
| EGFR | | | | |
| Negative | 18 | 31 | 12 | 0.09 |
| Positive | 6 | 31 | 12 | |
| Age | | | | |
| < 51 | 4 | 23 | 9 | 0.16 |
| > 50 | 20 | 39 | 15 | |

w, weak; +, positive; –, negative.

*Relationship of gp200-MR6 loss to survival*

A univariate analysis of overall survival showed that the most important prognostic factor was the nodal status ($P<0.0001$) followed by T-stage ($P=0.001$), grade ($P=0.09$, NS) and gp200-MR6 expression ($P=0.09$, NS). A multivariate

**Figure 2** Overall survival (**a**), local relapse-free survival (**b**) and distant metastasis-free survival (**c**) with respect to gp200-MR6 status in 110 breast cancer patients.

analysis showed that only T-stage and N-stage were independent prognostic factors. The hazard ratio of gp200-MR6-positive cases analysed for overall survival was 0.3 (CI = 0.07 – 1.30, $P = 0.1$). Twenty-two out of 24 (92%) patients with tumours showing strong gp200-MR6 staining are alive and 21 out of 24 were without disease up to the time of analysis. Sixty-five out of 86 (76%) weak/negative cases were alive at analysis. Adjuvant chemotherapy administration was equally distributed among gp200-MR6 groups ($P = 0.2$). Twenty-six out of 86 gp200-MR6-weak/negative cases received adjuvant chemotherapy compared with four out of 20 gp200-MR6-positive cases.

Figure 2a shows the Kaplan – Meier overall survival curves for three groups of gp200-MR6 staining. Strong positive staining was correlated with better survival, which was not significant on the log-rank test ($P = 0.10$). The local relapse-free survival was not different among groups ($P = 0.81$) (Figure 2b). The distant metastases-free survival was not significantly different, although it was better in cases with strong gp200-MR6 expression ($P = 0.18$) (Figure 2c). Analysis of gp200-MR6 expression in node-positive and -negative cases separately showed no effect on survival.

### Discussion

Elevated expression of IL-4R has been reported in a variety of tumour cell lines such as sarcomas and melanomas (Puri *et al.*, 1991; Obiri *et al.*, 1994). In a preliminary study, Al Jabaari *et al.* (1989) analysed the expression of MR6 on a variety of normal and malignant tissues. Normal tissues showed a negative or a weak positivity to MR6, and a variety of different types of tumour tissues analysed were found to be strongly positive. In that study, preliminary immunoscintigraphical data were also presented, suggesting that MR6 may prove to be useful for *in vivo* imaging and targeting immunotherapy.

More recently, we have reported in further detail on the expression of gp200-MR6 in lung (Tungekar *et al.*, 1991), colon (Kaklamanis *et al.*, 1992) and bladder (Tungekar *et al.*, 1996). In lung and colon, the expression of gp200-MR6 was found to decrease with malignancy, and 30% of squamous cell and adenocarcinoma lung cases were MR6-negative, whereas all ten small-cell lung carcinomas were negative. Positive staining was observed in 40 out of 44 colorectal carcinomas, whereas all bladder carcinoma samples retained their MR6 positivity. Thus, gp200-MR6 loss is associated with malignancy in some but not all tumour cell lineages. In breast cancer, it was found that approximately 50% of breast carcinomas (24% of *in situ*, 69% of invasive tumours) had reduced expression (Mat *et al.*, 1993).

In this study, we have examined the expression of gp200-MR6 in a large series of breast primary carcinomas to assess the distribution of this molecule in greater detail and to determine its significance in disease prognosis. Positive staining (strong and weak) was observed in 86 out of 110 (78%) cases, while 24 out of 110 (22%) exhibited strong positivity. This distribution of expression is in good agreement with a previous study (Mat *et al.*, 1993).

Menopausal status, tumour size, differentiation, nodal status and oestrogen receptors were not correlated with gp200-MR6 expression. These data suggest that gp200-MR6 status is not correlated with other known prognostic factors. A lower risk for distant metastases was observed in patients with positive gp200-MR6 expression. Taking into account our observations that MR6 stains normal breast, colon and lung epithelia, our data show that gp200-MR6 loss occurs during cancer evolution. As the IL-4 receptor is the target of IL-4 produced by NK cells in breast cancer patients (Lorenzen *et al.*, 1991), it seems likely that loss of the IL-4R-associated gp200-MR6 molecule could represent a mechanism of tumour escape from immune surveillance. IL-4R is also relevant for IL-4/toxin therapy (Debinski *et al.*, 1993) and IL-4 is used as a growth inhibitor. Like TGF, endogenous IL-4 may be inhibitory in normal tissues, and loss of IL-4 receptor or gp200-MR6 may be related to progression.

In a recent study, we showed an increased HLA class I antigenic loss in breast cancer metastatic to lymph nodes compared with the primary tumours (Kaklamanis *et al.*, 1995). It would be of interest to examine whether cells metastatic to lymph nodes or distant organs have a similarly higher occurrence of gp200-MR6 loss. Short-term distant metastasis-free survival was better in patients with MR6-positive tumours. The combined role of HLA class I and IL-4R/gp200-MR6 in survival of breast cancer patients requires further investigation.

Although expression of gp200-MR6 on normal epithelia and haemopoietic cells raises potential problems of toxicity for the use of radiolabelled and toxin-conjugated antibody and cytokine targeting (Al Jabaari *et al.*, 1989; Debinski *et al.*, 1993), the differences in IL-4R structure on these different cell types may negate such problems (Murata *et al.*, 1996; Bamborough *et al.*, 1993). Finally, although there are many prognostic factors in breast cancer, none of them relate to immunological pathways; the data presented in this study suggest the potential of lymphokines as an independent pathway regulating cancer growth.

### References

AL JABAARI B, LADYMAN HM, LARCHE M, SIVOLAPENCO GB, EPENETOS AA AND RITTER MA. (1989). Elevated expression of the interleukin-4 receptor in carcinoma: a target for immunotherapy? *Br. J. Cancer*, **59**, 910 – 914.

BAMBOROUGH P, GRANT GH, HEDGECOCK CJ, WEST SP AND RICHARDS WG. (1993). A computer model of the interleukin-4/receptor complex. *Protein*, **17**, 11 – 19.

CORDELL JL, FALINI B, ERBER W, GHOSH A, ABDULAZIZ Z, MACDONALD S, PULFORD KAS, STEIN H AND MASON DY. (1984). Immunoenzymatic labelling of monoclonal antibodies using immune complexes of alkaline phosphatase and monoclonal anti-alkaline phosphatase (APAAP). *J. Histochem. Cytochem.*, **32**, 219.

DEBINSKI W, PURI RK, KRETMAN RJ AND PASTAN I. (1993). A wide range of human cancers express interleukin 4 (IL4) receptors that can be targeted with chimeric toxin composed of IL4 and Pseudomonas exotoxin. *J. Biol. Chem.*, **268**, 14065 – 14070.

DE MAAGD RA, MACKENZIE WA, SCHUURMAN H-J, RITTER MA, PRICE KM, BROEKHUIZEN R AND KATER L. (1985). The human monoclonal antiepithelial cell antibodies. *Immunology*, **54**, 745 – 754.

EORTC BREAST COOPERATIVE GROUP. (1980). Revision of the standards for the assessment of hormone receptors in human breast cancer. *Eur. J. Cancer*, **16**, 1313 – 1315.

FRAKER PJ AND SPECK JC. (1978). Protein and cell membrane iodinations with a poorly soluble chloramide 1,3,4,6-tetrachloro-3 a, 6 a-diphenyl-glycolucil. *Biochem. Biophys. Res. Commun.*, **80**, 849 – 857.

GOLUMBEK PT, LAZENBY AJ, LEVITSKY HI, JAFFE LM, KARASUYAMA H, BAKER M AND PARDOLL DM. (1991). Treatment of established renal cancer by tumor cells engineered to secrete interleukin-4. *Science*, **254**, 713 – 716.

HOON DS, OKUN E, BANEZ M, IRIE RF AND MORTON DL. (1991). Interleukin 4 alone and with gamma interferon or alpha tumor necrosis factor inhibits cell growth and modulates cell surface antigens on human renal cell carcinomas. *Cancer Res.*, **51**, 5687 – 5693.

HOWARD M, FAZZAR J, HIFFIKER M, JOHNSON B, TAKATSU K, HAMAOKA T AND PAUL WE. (1982). Identification of a T-cell derived B-cell growth factor distinct from Interleukin-2. *J. Exp. Med.*, **155**, 914 – 923.

IZUHARA K, YANG G AND MIYAJIMA A. (1993). Structure of the IL-4 receptor and signal transduction mechanism of IL-4. *Res. Immunol.*, **144**, 584 – 590.

IMAMI N, LARCHE M AND RITTER MA. (1994). Inhibition of alloreactivity by mAb MR6: differential effects on IL-2- and IL-4 producing human T cells. *Int. Immunol.*, **6**, 1575 – 1584.

KAKLAMANIS L, GATTER KC, MORTENSEN N AND HARRIS AL. (1992). Interleukin-4 receptor and epidermal growth factor receptor expression in colorectal cancer. *Br. J. Cancer*, **66,** 712–716.

KAKLAMANIS L, LEEK R, KOUKOURAKIS M, GATTER KC AND HARRIS AL. (1995). Loss of TAP-1 transport protein and MHC-class I molecules in metastatic compared to primary breast cancer. *Cancer Res.*, **55,** 5191–5194.

LARCHE M, LAMB JR, O'HEHIR RE, IMAMI-SHITA N, ZANDERS E AND RITTER MA. (1988). A monoclonal antibody recognising the human interleukin-4 receptor. *Immunology*, **65,** 617–622.

LORENZEN J, LEWIS CE, MCCRACKEN D, HORAK E, GREENAL M AND MCGEE JOD. (1991). Human tumor-associated NK cells secrete increase amounts of interferon-gamma and interleukin-4. *Br. J. Cancer*, **64,** 457–462.

MAT IB, MOORS N, MELCHER D, FOXWELL BMJ AND RITTER MA. (1993). Comparison of MR6-Ag/IL-4 receptor complex and c-erB-2 antigen expression in breast tumours. In *Mutant Oncogenes, Targets for Therapy*, pp. 53–64. Chapman & Hall: London.

MAT I, LARCHE M, MELCHER D AND RITTER MA. (1990). Tumour-associated up-regulation of the IL-4 receptor complex. *Br. J. Cancer*, **62,** Suppl. x, 96–98.

MONROE JG, HALDER S, PRYSTOWSKY MB AND LAMMIE P. (1988). Lymphokine regulation of inflammatory process: interleukin 4 stimulates fibroblast proliferation. *Clin. Immunol. Immunopathol.*, **49,** 292.

MURATA T, NOGUCHI PD AND PURI RK. (1996). Receptors for interleukin (IL)-4 do not associate with the common Á chain, and IL-4 induces the phosphorylation of Jak2 tyrosine kinase in human colon carcinoma cells. *J. Biol. Chem.*, **270,** 30829–30836.

NICHOLSON S, SAINSBURY JRC, NEEDHAM GK, CHAMBERS P, FARDON JR AND HARRIS AL. (1988). Quantitative assays of epidermal growth factor receptor in human breast cancer: cut-off points of clinical relevance. *Int. J. Cancer*, **42,** 36–41.

OBIRI NI, SIEGEL JP, VARRICCHIO F AND PURI RK. (1994). Expression of high affinity receptors on human melanoma, ovarian and breast carcinoma cells. *Clin. Exp. Immunol.*, **95,** 148–155.

O'HARA J AND PAUL WE. (1987). Receptors for B-cell stimulatory factor-1 expressed on cells of haemopoietic lineage. *Nature*, **325,** 537.

OHIRA T, OHE Y AND SAIJO N. (1992). Induction of tumor immunity by cytokine cDNA transfected Lewis lung carcinoma. *Nippon Kyobu Shikkan Gakkai Zasshi*, **30**(suppl.), 48–51.

PURI RK, OGATA M, LELAND P, FELDMAN GM, FITZGERALD D AND PASTAN I. (1991). Expression of high affinity interleukin 4 receptors on murin sarcoma cells and receptor mediated cytotoxicity of tumor cells to chimeric protein between interleukin 4 and Pseudomonas exotoxin. *Cancer Res.*, **51,** 3011–3017.

THORNHILL MH AND HASKARD DO. (1990). IL-4 regulates endothelial cell activation by IL-1, tumor necrosis factor or IFNg. *J. Immunol.*, **145,** 865–872.

TOI M, HARRIS AL AND BICKNELL R. (1991). Interleukin-4 is a potent mitogen for capillary endothelium. *Biochem. Biophys. Res. Commun.*, **174,** 1287–1293.

TOI M, BICKNELL R AND HARRIS AL. (1992). Inhibition of colon and breast carcinoma cell growth by IL-4. *Cancer Res.*, **52,** 275–279.

TUNGEKAR G, TURLEY H, DUNNIL MS, GATTER KC, RITTER MA AND HARRIS AL. (1991). Interleukin-4 receptor expression on human lung tumors and normal lung. *Cancer Res.*, **51,** 261–264.

TUNGEKAR MF, GATTER KC AND RITTER MA. (1996). Bladder carcinomas and normal urothelium universally express gp200-MR6, a molecule functionally associated with the interleukin-4 receptor (CD124). *Br. J. Cancer*, **73,** 429–432.

TEPPER RI, PATTENGALE PK AND LEDER P. (1989). Murine interleukin-4 displays potent anti-tumor activity in vivo. *Cell*, **57,** 503–512.

TOTPAL K AND AGGARWAL BB. (1991). Interleukin 4 potentiates the antiproliferative effects of tumor necrosis factor on various tumor cell lines. *Cancer Res.*, **51,** 4266–4270.

VON-GAUDECKER B, LARCHE M, SCHUURMAN HJ AND RITTER MA. (1989). Analysis of the fine distribution of thymic epithelial microenvironmental molecules by immuno-electron microscopy. *Thymus*, **13,** 187–194.

ZALOOM Y AND GALLAGHER G. (1993). IL-2 inhibits the induction of systemic antitumour immunity by IL-4 in the peritumoral treatment of experimental melanoma. *Anticancer Res.*, **13,** 1081–1085.